# Open Eyes Increase Neural Oscillation and Enhance Effective Brain Connectivity of the Default Mode Network: Resting-State Electroencephalogram Research

**DOI:** 10.3389/fnins.2022.861247

**Published:** 2022-04-27

**Authors:** Yi Wang, Jialu Li, Lingjing Zeng, Haiteng Wang, Tianyi Yang, Yongcong Shao, Xiechuan Weng

**Affiliations:** ^1^Department of Physical Education, Renmin University of China, Beijing, China; ^2^School of Life Sciences and Technology, Harbin Institute of Technology, Harbin, China; ^3^School of Psychology, University of Leeds, Leeds, United Kingdom; ^4^School of Psychology, Beijing Sport University, Beijing, China; ^5^Beijing Institute of Basic Medical Sciences, Beijing, China

**Keywords:** default mode network, resting-state EEG, neural oscillation, effective connectivity, mood

## Abstract

The default mode network (DMN) has a unique activity pattern in the resting brain. Studies on resting-state brain activity are helpful to identify various brain dynamic characteristics of patients with mental diseases and those of healthy people. The brain produces a series of changes in different eye states. However, the relationship between eye states and the DMN, which is closely related to the resting state, has not been widely examined. This study recruited 42 healthy students aged 17–22. Participants completed the Profile of Mood States questionnaire. Thereafter, the electroencephalogram data was collected with the patients’ eyes open and closed. Changes in neural oscillation and the DMN’s information transmission during different eye openness states were compared. The results showed that the neural oscillation activities of the parietal-occipital network such as the superior parietal lobule and precuneus were significantly enhanced in the eyes open state. In addition, the effective connectivity within the DMN was enhanced during opened eyes, especially from the left precuneus to the left posterior cingulate cortex, and this connectivity was negatively correlated with the Vigor-Activity mood state in the eyes open state. The activity of the DMN in the resting-state is regulated by eye states, which may relate to mood and emotional perception.

## Introduction

Rest is characterized by a constant state without stimulation or other significant behavior, whether eyes are open or closed, with or without visual fixation (Snyder and Raichle, 2012). Resting state electrophysiological and neuroimaging studies are considered to provide neurophysiological evidence for the early identification of patients with mental diseases and Alzheimer’s disease (Blackburn et al., 2018; Babiloni et al., 2020; Büyükgök et al., 2020). Previous studies have shown that voluntary eye opening or closing can regulate brain activity and connectivity (Costumero et al., 2020), and the statistical characteristics of endogenous activity are also regulated by eye states [eyes open, (EO); eyes closed, (EC)] (Bianciardi et al., 2009). For example, a resting-state functional magnetic resonance imaging (fMRI) study indicated significant differences in the functional connectivity between EO and EC, mainly reflected in the thalamus’s amplitude modulation of low-frequency spontaneous activities of the sensory system (Qian et al., 2019). One resting-state electroencephalogram (EEG) study found that there were significant differences in brain activities between eye states, mainly reflected in increasing α and β interhemispheric coherence with the EO state in frontal, temporal and occipital lobes (Gorantla et al., 2020). Currently, the number of resting-state EEG studies has increased sharply. This may be conducive to understanding the dynamic characteristics of the brain (Gómez-Ramírez et al., 2017) and may aid in effectively distinguishing healthy people from patients with mental diseases, such as epilepsy, by studying the dynamic characteristics of the brain during different eye states (Hussain et al., 2017). However, due to the diversity of brain research techniques and methods, previous studies on the changes of resting-state brain activity in healthy people with different eye states are insufficient to form a coherent view.

Researchers often use time-domain and frequency-domain methods such as independent component analysis (ICA), EEG micro-state and low-frequency θ-β ratio to compare the brain neural activities of individuals with EO and EC during resting state (Gorantla et al., 2020; Tamburro et al., 2021). In studies that directly compare the difference between EO and EC during resting state, EEG research accounts for a relatively small proportion. EEGs with a millisecond time resolution are powerful and can be used to check the activity of brain networks across the cortex (Khanna et al., 2015). As a simple and high-precision brain imaging analysis method (Wagner et al., 2004), exact low resolution brain electromagnetic tomography (eLORETA) can compare the changes of neural oscillations in resting state brain networks, such as using a functional independent component analysis (fICA) (Aoki et al., 2015). It also allows to compare changes of information flow between each brain region through an isolated effective correlation analysis (iCoh) (Fernandez Guerrero and Achermann, 2018). This method of analysis can provide a possibility for further study of the dynamic changes of different resting-state eye states. Therefore, the purpose of this study was to compare the changes in brain neural oscillation and effective connectivity during the EO and EC states, by EEG based on fICA and iCoh methods.

The default mode network (DMN), is a large-scale network and mainly includes the medial prefrontal cortex, posterior cingulate cortex, inferior parietal lobule, and precuneus (Greicius et al., 2009; Shirer et al., 2012). Activity in the DMN is considered to increase in the resting-state, while the activation of this brain region decreases when performing specific tasks (Aoki et al., 2015). Several fMRI studies found that there were significant differences in functional connectivity between different eye states during resting state, mainly reflected in the connectivity between the sensory motor network and the DMN, and the thalamus’ regulation of the sensory system (Wang et al., 2015; Qian et al., 2019). A study indicated that the EEG spectrum showed different characteristics during EO and EC states, that is, the delta and theta power spectra are enhanced during the EC state (Allen et al., 2018). Eye states can regulate brain information processing, which may relate to changes in DMN activity. Therefore, the DMN may play a vital role in interpreting the results of research on brain dynamics during different eye states. This is the first study to use fICA and iCoh methods to probe the relationship between eye states and the DMN, which provides a method and theoretical possibility to explore the dynamic changes of the DMN’s neural oscillation and effective connectivity under different eye states during resting state.

This study also aimed to explore the changes in the DMN’s neural oscillation and effective connectivity in healthy people with different eye states and the relationship between brain activity and mood state, to better understand the dynamic characteristics of the resting brain. Previous studies have found that different eye states can produce measurable changes in the DMN (Yan et al., 2009; Liang et al., 2014), which might be related to multisensory system, perception, as well as mood state (Wang et al., 2015; Renner et al., 2017). Based on the findings of these previous studies, we hypothesized that low-frequency neural oscillation of the DMN and effective connectivity within the DMN would be affected by different eye states, which might correlate with the mood state.

## Materials and Methods

### Participants

Forty-two university students aged from 17 to 22 years (mean age, 19.79 ± 1.24 years; gender proportion, 1:1) were recruited from Beijing Sport University. All participants were right-handed, with normal uncorrected or corrected vision, no tactile perception disorder related diseases, and had not participated in psychological and physiological tests before. Besides, none of the participants had any neurological or psychiatric disorders. This study was approved by the ethics committee of Beijing Sport University, and the participants signed a written informed consent before the experiment.

### Resting-State Electroencephalogram Task

The setup of the EEG headset took 18 min, including soaking it in a potassium chloride (KCl) solution for 15 min and wearing the headset for 3 min to ensure that the impedance of each electrode was <50 kΩ. After being comfortably seated, participants were asked to remain quiet for 2 min and to relax. During EEG data acquisition, the conditions were counterbalanced. Participants were asked to maintain the EO/EC state first, and then there was a 2-min interval before the EC/EO state. Twenty-one participants maintained the EO state first, and the other 21 participants experienced the EC state first. Data acquisition for each condition took 5 min. EEG data acquisition took a total of 32 min.

### Electroencephalogram Recording

High-density EEG was recorded using an electrode net (Geodesic Sensor Net, Electrical Geodesics Inc., Eugene, OR, United States) comprising 256 electrodes interconnected with thin rubber bands, and containing small sponges soaked with saline water, which were in direct contact with each participant’s scalp surface.

Electroencephalogram data were acquired with a 256-channel HydroCel Geodesic Sensor Net (EGI, Eugene, OR) using Net Station 4.5 software. All electrode impedances were below 50 kΩ before the recording was started, and the band filters were applied between 0.1 and 40 Hz during a continuous recording, which included a 50 Hz notch filter to remove power supply noise. EEG recordings were sampled at 1000 Hz.

### Profile of Mood States

The Chinese version of the POMS questionnaire was used in our study (Wu and Zou, 2020). This questionnaire has six scales (i.e., Tension-Anxiety, TA; Depression-Dejection, DD; Anger-Hostility, AH; Fatigue-Inertia, FI; Confusion-Bewilderment, CB; Vigor-Activity, VA) with a total of 65 items. Each item was assessed by the participants in a five-point Likert scale (from “1-not at all” to “5-extremely”). This test has a simple list of emotional adjectives, which allows people to match their current emotions. Participants were asked to assess their emotions before conducting EEG tests.

## Data Analysis

### Electroencephalogram Data Preprocessing

Preprocessing of EEG data was performed using the toolbox EEGLAB in MATLAB 2015b software. There were seven main steps to screen the EEG data: (1) Seventy electrodes of the international 10–20 system were selected; (2) The sampling rate was downsampled to 500 Hz; (3) The infinite impulse response Butterworth band-pass filtering with bandpass of 0.1–40 Hz and frequency slope of 24 dB/oct was performed; (4) The whole brain average reference was used for re-reference; (5) The ICA was used to eliminate the components with obvious artifacts; 6) The plug-in ADJUST 1.1.1 in EEGLAB was used to semi-automatically remove independent components with eye movement related obvious artifacts; (7) Data containing obvious drift or artifacts were removed by the plug-in Clean Rawdata in EEGLAB. Artifact-free EEG data were then retained.

### Functional Independent Component Analysis

The fICA method in eLORETA was undertaken to compare changes in brain neural oscillations between EO and EC states, within the same brain network (Aoki et al., 2015). We calculated the electrocortical activity of the brain network according to the following frequency bands: delta, 1–4 Hz; theta, 4–8 Hz; alpha, 8–13 Hz; and beta, 13–30 Hz. The intuitive and exploratory method took each distinct pair of electrodes, and computed the correlation between the signals. If two signals were highly correlated, coherent, and synchronized, then they were assigned to the same network. If two signals were dissimilar, they were assigned to different networks. The brain was automatically divided into seven networks using this algorithm (i.e., seven components of interest), and each data representing each participant could generate seven coefficients (one coefficient for each network, expressing how that participant used that network). Subsequently, statistical analysis was performed on these seven numbers, comparing the differences in resting-state networks when eyes were open compared to when eyes were closed. Subsequently, an independent sample *t*-test was used to compare the coefficients of different eye states. The statistical non-parametric mapping (SnPM) method (Thatcher et al., 2005) was used to determine differences in the resting state networks between EO and EC states by paired *t* tests, using low resolution brain electromagnetic tomography (LORETA) software, and the SnPM method was used to correct for multiple comparisons (Tang et al., 2013; Painold et al., 2020). Finally, the brain networks with significant differences were found, and the coefficient of the significant network was derived, which was termed the fICA value. The fICA method has been described by Aoki et al. (2015).

### Effective Connectivity Analysis

Effective connectivity was calculated by the iCoh method (Pascual-Marqui et al., 2014), which aimed to access direct paths of intracortical causal information flow of oscillatory activity. Considering previous research and the intention of our study (Liu et al., 2020), brain regions belonging to the DMN were selected as regions of interest (ROIs) in our study (Table 1): middle frontal gyrus (Brodmann Area [BA] 11), posterior cingulate cortex (BA31), precuneus (BA7), inferior parietal lobule (BA40), and middle temporal gyrus (BA21). We aimed to analyze the effective connectivity within the DMN and their changes between EO and EC conditions. The iCoh was used to calculate the iCoh value of the effective connectivity within the DMN from 1 Hz to 30 Hz (four frequency bands: delta, theta, alpha, and beta). The iCoh values of two conditions were compared through the statistics function. During the *t*-test, the randomization SnPM was performed (number of randomizations = 5000), and the corrected critical thresholds and *p* values were computed. Then the threshold [t] values representing the difference of effective connectivity within the DMN were obtained. Finally, the results were read through the PlotFunc function.

**TABLE 1:** The Coordinates of ROIs in the DMN.

Regions of interest	Hemisphere	x	y	z	BA
Middle frontal gyrus	L	−35	40	−20	11
Middle frontal gyrus	R	35	40	−20	11
Posterior cingulate cortex	L	−5	−60	30	31
Posterior cingulate cortex	R	5	−60	30	31
Precuneus	L	−10	−65	65	7
Precuneus	R	10	−65	65	7
Inferior parietal lobule	L	−45	−45	50	40
Inferior parietal lobule	R	45	−45	50	40
Middle temporal gyrus	L	−50	5	−40	21
Middle temporal gyrus	R	50	5	−40	21

*ROIs, regions of interest; DMN, default mode network; L, left; R, right; xyz = coordinates of the MNI space for each ROI; BA, Brodmann area.*

### Statistical Analyses

SPSS 25.0 software was used to perform the Pearson’s correlation analysis on the effective connectivity changes with significant differences between each eye state (EO and EC) and each POMS score (overall, TA, DD, AH, FI, CB, and VA scores, respectively). To eliminate the familywise error rate, a Bonferroni correction was used for multiple comparisons.

## Results

### Neural Oscillation During Eyes Open and Eyes Closed States

**FIGURE 1:**
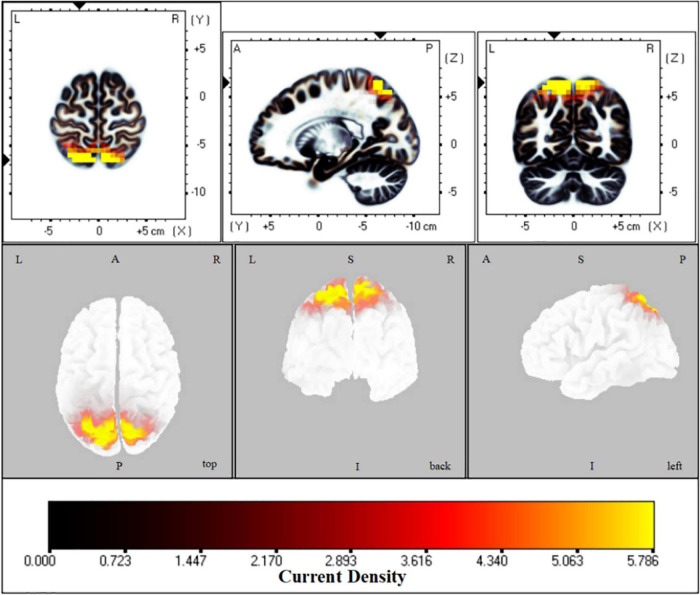
Functional independent component analysis (flCA): Neural oscillation changes in the resting state. Changes of information transmission in the left superior parietal lobule between eyes open and eyes closed states in delta band; The bright yellow indicates increased delta oscillation in the eyes open state. L, left; A, anterior; R, right; P, posterior; S, superior; I, inferior.

The fICA results indicated that in two eye states the parietal-occipital network was significantly different (*t* = 1.6, *p* < 0.01). Compared with an EC state, the activation of the superior parietal lobule, precuneus and postcentral gyrus increased in EO in all frequency bands, especially in the delta band (*t* = 7.23) (Figure 1).

### Effective Connectivity of the Default Mode Network

**FIGURE 2:**
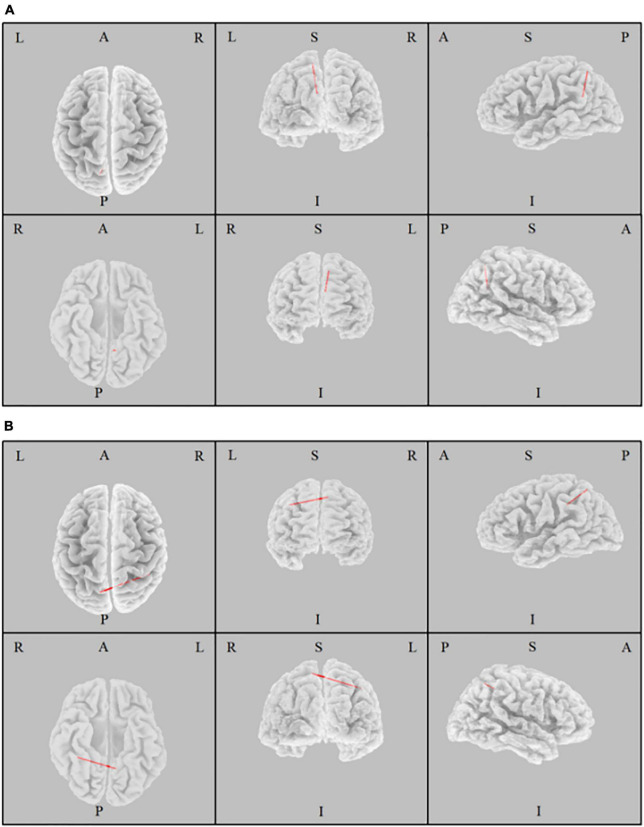
The Change of Effective Connectivity between eyes open and eyes closed states. The effective connectivity from the left precuneus to the left posterior cingulate cortex **(A)** and to the right inferior parietal lobule **(B)** of beta band was significantly increased in the eyes open state compared with the eyes closed state.

The iCoh analysis found that there were significant differences in the connectivity within the DMN between two eye states (*t* = 3.4, *p* = 0.023). In the beta band, the effective connectivity from the left precuneus to the left posterior cingulate cortex was abbreviated as beta-PC, and the effective connectivity from the left precuneus to the right inferior parietal lobule was abbreviated as beta-PI. We found that effective connectivity of beta-PC and beta-PI was significantly enhanced in the EO state compared with the EC state (Figure 2). Therefore, the effective connectivity of beta-PC and beta-PI was considered in the subsequent correlation analysis.

### Correlation Between Effective Connectivity Changes and Mood State Changes

**FIGURE 3:**
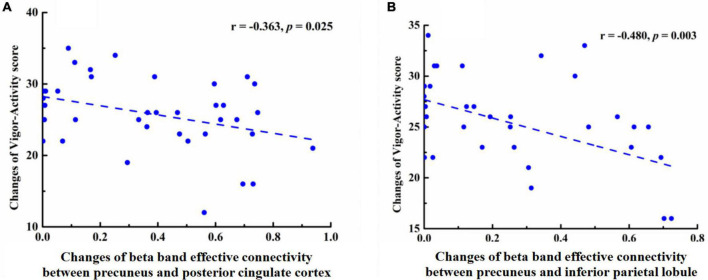
Pearson correlation coefficient between changes in effective connectivity and changes in Vigor scores, **(A)** A negative correlation was found between beta-PC effective connectivity and Vigor score in the eyes open state (r = −0.363, p = −0.025). **(B)** A negative correlation was found between beta-PI effective connectivity and Vigor score in the eyes open state (r = −0.480, p = −0.003).

Pearson’s correlation analysis found that in the EO state, the effective connectivity of beta-PC (*r* = −0.363, *p* = 0.025) and beta-PI (*r* = −0.480, *p* = 0.003) were both negatively correlated with the VA score (Figure 3). Meanwhile, in the EC condition, the effective connectivity of beta-PC was positively correlated with the TA score (*r* = 0.378, *p* = 0.025) and DD score (*r* = 0.355, *p* = 0.037). However, no significant correlation between the effective connectivity of beta-PI and mood states was found in our study. After the Bonferroni correction, only the negative correlation between the beta-PI and VA score was still statistically significant, with a *p* value less than 0.004 (i.e., 0.05/14 = 0.004).

## Discussion

This study examined the relationship between eye states and resting-state brain activity in healthy university students. The results further showed that there were significant differences in the dynamic characteristics of the brain when the eyes were opened or closed. In the EO state, the neural oscillation of the superior parietal lobule, precuneus and postcentral gyrus were significantly enhanced in the delta frequency band, and the effective connectivity of multiple brain regions in the DMN was enhanced. Additionally, changes in the DMN information flow were manifested in the enhanced connectivity in the EO state from the left precuneus to the left posterior cingulate cortex and the right inferior parietal lobule. Interestingly, this study found that the enhanced effective connectivity during the EO state was negatively correlated with a positive mood state (i.e., VA).

Similar to previous studies, we found that there was a consistent enhancement in the neural oscillation of the DMN in the resting-EO state (Yan et al., 2009; Chen et al., 2013; Mo et al., 2013). Numerous studies have shown that brain activity in the resting state is regulated by alpha rhythm (Kay et al., 2012; Clancy et al., 2020), and some studies have pointed out that high-frequency activity is usually related to cognitive and task processing (Deligianni et al., 2014; Fries, 2015; Sockeel et al., 2016). However, this study further found that the enhancement of the DMN activity during eye opening occurred in a wide frequency band, which concurred with results of previous studies (Wang et al., 2019). Compared with the global efficiency in the EC state, the global efficiency of the dynamic state in the EO state is higher, which is considered the reorganization of the brain network in the state of external perception (Wang et al., 2015). Therefore, our findings indicated that enhanced brain activity shows efficient organization of the brain network in the EO state, which is closely related to information transmission about the external environment (Jin et al., 2014). It may also be that compared with the EC state, the overall spontaneous activity of the brain is higher, and there may be mind-wandering and daydreaming with the EO (Yan et al., 2009), resulting in significant high-frequency activity.

Our study found that compared with the EC state, the effective connectivity within the DMN was enhanced in the resting-EO state, which was mainly reflected in increased communications between the precuneus and posterior cingulate cortex/inferior parietal lobule; this differed from the results of previous studies (Patriat et al., 2013; Liu et al., 2020). Prior studies claimed that there was no significant difference in the connectivity within the DMN during different eye states in healthy people. This may be because the fMRI technique with high spatial resolution is used most to research brain connectivity (Horwitz, 2003). Moreover, the fMRI calculates functional connectivity through signal fluctuations related to blood-oxygen-level-dependent imaging (Biswal et al., 1995), which differs from calculating the effective connectivity of the information flow changes by neural oscillation (Massimini et al., 2005). Liu et al. (2020) found that different eye states in the resting state may induce different time-varying neural activities in the DMN by a sliding-window approach. However, in the same situation, another fMRI study found that there were significant differences in brain activities, mainly reflected in the dynamic spontaneous functional connectivity of the brain (Wang et al., 2015). These varied results may be because of the selection of ROI and the calculation method of brain connectivity (Massimini et al., 2005; Liu et al., 2020).

Interestingly, this study found that there was a significant negative correlation between beta-PI and VA during different eye states. The effect of eye states on brain activity is considered to be related to attention and emotions (Chang et al., 2015). This concurs with results of previous studies, that is, brain connectivity is negatively correlated with positive emotion states. However, in these studies, the ROI is a large network, including the medial prefrontal cortex, thalamus, superior temporal sulcus and posterior cingulate/precuneus (Rohr et al., 2013). Changes in the connectivity within the DMN are considered to be related to self-awareness and negative emotions (Lancaster et al., 2019; Zhang and Volkow, 2019). In the field of depression, the increase of connectivity or low-frequency power within the DMN is considered to be mediated by mood disorders (Ho et al., 2015; Hwang et al., 2016; Slobodskoy-Plusnin, 2018). However, this study found that the change in information transmission within the DMN in the high-frequency beta is more likely to be related to the stable positive mood state than the emotion induced by stimulation.

This study was limited in several ways. Firstly, there is only one sub-scale relative to positive emotion in the POMS questionnaire (i.e., VA sub-scale), which makes it difficult to evaluate individual positive mood states thoroughly. Secondly, because there are many noise artifacts in 256 channels, this study only selected 70 electrodes in the international 10–20 system for analysis, which reduced the spatial resolution and led to a decline in the accuracy of data results. In addition, this study collected EEG data during resting state, so it was impossible to draw any conclusion about the relationship between task-induced positive/negative emotions and brain activity of the DMN during resting state. Moreover, this study focused on the regulation of eye states on the effective connectivity within the DMN, but the effect of eye states may be related to broader brain networks, such as emotional and salience networks. Last, it is challenging to separate DMN from other resting state networks, and there may be partial overlap between brain networks. Therefore, more studies are needed to explore the relationship between mood state, emotional stimulation, and brain network during different eye states through neuroimaging technology with a higher spatial resolution, such as fMRI and positron emission tomography.

This unique study explored the relationship between eye state and effective connectivity within the DMN. The study found that the effective connectivity within the DMN was enhanced when the eyes were open, which was specifically reflected in information transmission from the precuneus to the inferior parietal lobule; this connectivity was significantly correlated with a positive mood state. Moreover, the results show that different eye states can be recognized through the changes of the high-frequency connectivity within the DMN during resting state. Additionally, the relationship between DMN connectivity and emotion suggests that emotion perception may be regulated by the eye state. Further research is needed to obtain a better understanding of the relationship between eye state and emotion perception through the DMN and other brain network activities.

## Data Availability Statement

The raw data supporting the conclusions of this article will be made available by the authors, without undue reservation.

## Ethics Statement

The studies involving human participants were reviewed and approved by the Ethics Committee of Beijing Sport University. The patients/participants provided their written informed consent to participate in this study.

## Author Contributions

YS and XW contributed to the design of the experiments. YS and YW collected experimental data. YW and JL obtained the results and wrote the manuscript. LZ, HW, and TY analyzed and interpreted the data. All authors listed have read and approved the final manuscript.

## Conflict of Interest

The authors declare that the research was conducted in the absence of any commercial or financial relationships that could be construed as a potential conflict of interest.

## Publisher’s Note

All claims expressed in this article are solely those of the authors and do not necessarily represent those of their affiliated organizations, or those of the publisher, the editors and the reviewers. Any product that may be evaluated in this article, or claim that may be made by its manufacturer, is not guaranteed or endorsed by the publisher.
